# State and Regional Variation in Prescription- and Payment-Related Promoters of Adherence to Blood Pressure Medication

**DOI:** 10.5888/pcd17.190440

**Published:** 2020-09-24

**Authors:** Peter K. Yang, Matthew D. Ritchey, Stavros Tsipas, Fleetwood Loustalot, Gregory D. Wozniak

**Affiliations:** 1 Division for Heart Disease and Stroke Prevention, National Center for Chronic Disease Prevention and Health Promotion, Centers for Disease Control and Prevention, Atlanta, Georgia; 2 Oak Ridge Institute for Science and Education, Oak Ridge, Tennessee; 3 Improving Health Outcomes Group, American Medical Association, Chicago, Illinois

## Abstract

**Introduction:**

Medication adherence can improve hypertension management. How blood pressure medications are prescribed and purchased can promote or impede adherence.

**Methods:**

We used comprehensive dispensing data on prescription blood pressure medication from Symphony Health’s 2017 Integrated Dataverse to assess how prescription- and payment-related factors that promote medication adherence (ie, fixed-dose combinations, generic formulations, mail order, low-cost or no-copay medications) vary across US states and census regions and across the market segments (grouped by patient age, prescriber type, and payer type) responsible for the greatest number of blood pressure medication fills.

**Results:**

In 2017, 706.5 million prescriptions for blood pressure medication were filled, accounting for $29.0 billion in total spending (17.0% incurred by patients). As a proportion of all fills, factors that promoted adherence varied by state: fixed-dose combinations (from 5.8% in Maine to 17.9% in Mississippi); generic formulations (from 95.2% in New Jersey to 98.4% in Minnesota); mail order (from 4.7% in Rhode Island to 14.5% in Delaware); and lower or no copayment (from 56.6% in Utah to 72.8% in California). Furthermore, mean days’ supply per fill (from 43.1 in Arkansas to 63.8 in Maine) and patient spending per therapy year (from $38 in Hawaii to $76 in Georgia) varied. Concentration of adherence factors differed by market segment. Patients aged 18 to 64 with a primary care physician prescriber and Medicaid coverage had the lowest concentration of fixed-dose combination fills, mean days’ supply per fill, and patient spending per therapy year. Patients aged 65 years or older with a primary care physician prescriber and commercial insurance had the highest concentration of fixed-dose combinations fills and mail order fills.

**Conclusion:**

Addressing regional and market segment variation in factors promoting blood pressure medication adherence may increase adherence and improve hypertension management.

**SUMMARY**

**What is already known on this topic?**

Approximately three-fourths of US adults with hypertension do not have their blood pressure controlled. Medication adherence is important in hypertension management and can be affected by how medications are prescribed and purchased.

**What is added by this report?**

We found considerable variation in prescription- and payment-related factors that promote medication adherence by geography and across the largest patient market segments comprised of medication prescriber, insurance payer type, and age.

**What are the implications for public health practice?**

Blood pressure control rates are low and may be affected by uptake of the adherence promotion factors assessed. Increased uptake of these promoters, especially in the regions and populations in most need, could improve hypertension management.

## Introduction

Hypertension is highly prevalent in the United States, affecting almost half of US adults (1). In most cases, hypertension can be effectively managed through lifestyle modification and often with pharmacologic therapy (2,3). However, around three-fourths of US adults with hypertension have blood pressures (BPs) above the thresholds recommended in current guidelines, placing them at increased risk for heart disease and stroke (2).

Medication nonadherence, defined as patients not taking medication as prescribed by their health care provider, is a modifiable barrier to effective management of hypertension and other chronic diseases. Nonadherence increases US health care costs by hundreds of billions of dollars annually (4), often because of the increased risk for cardiovascular events such as myocardial infarction and stroke (4). BP medication nonadherence is highly prevalent and varies by geography and patient demographics (5,6). Multiple prescription- and payment-related factors have been identified that can improve BP medication adherence, thereby increasing the number of patients who achieve a BP goal (7). Prescription-related factors are prescribing fixed-dose combination medications to reduce total pill consumption (8), using mail order prescriptions to address barriers in access to retail pharmacies and to make acquiring prescriptions more convenient for patients (9), and increasing the days’ supply per fill to decrease pharmacy visits (10). Payment-related factors to reduce financial barriers are prescribing low-price generic formulations (11), using medications with lower or no patient copayments (12), and minimizing overall out-of-pocket costs for patients (13).

Previous research has described national trends in prescription- and payment-related factors that promote improved BP medication adherence (14). However, we are unaware of any study assessing state and regional variation in these factors, especially by market segment. Therefore, we used data representing most prescription BP medications filled from US retail and mail order pharmacies in 2017 to describe geographic variation in these adherence promotion factors across the largest market segments (ie, combinations of prescriber type and primary insurance payer type), by patient age group, and by US Census region. These findings can inform strategies to improve BP medication adherence and hypertension control.

## Methods

We obtained prescription fill data through Symphony Health’s 2017 Integrated Dataverse (IDV) (15). The IDV contains data on over 90% of outpatient prescription fills from retail and mail order pharmacies, and combined with market purchasing data, creates national fill and spending estimates. Symphony Health provided data on aggregate number of fills, therapy days, and spending, including total spending and patient spending, for BP medication. These data are presented by 3 patient demographics (age group [18–64 y or ≥65 y], US Census region [northeast, midwest, south, west], and the state the prescription was prescribed in); by 4 prescriber specialties: primary care physicians (PCPs) (includes family practice, internal medicine, and osteopathic medicine], cardiologists, nurse practitioners and physician assistants, and other physician prescribers); 2 pharmacy types (mail order and retail); 2 formulation types (brand and generic); and 4 primary payer types (patient out-of-pocket, commercial insurance, Medicare Part D, and Medicaid). Fills that contained more than one BP-lowering medication per pill (ie, fixed-dosed combinations) were counted by the total number of drugs contained when determining the total number of medications filled and total therapy years (1 therapy year equals 365 days of available medication) of BP medication dispensed.

Descriptions of adherence promotion factors related to prescriptions and payment used in our study are available elsewhere (14). Briefly, prescription-related factors are the percentage of fills that were for fixed-dose combination, the percentage that were fills by mail order, and the mean number of days’ supply per fill, defined as the length of time before a prescription would need to be refilled. Payment-related factors are the percentages of fills for generic formulations and fills with lower or no patient copayment ($5.00 or less per fill), and patient spending per years’ supply of medication (estimated cost of having medication on hand for 365 days). To account for missing values in patient spending (2.6% of fills), we calculated patient spending-value means stratified by medication class (eg, β-blockers) and payer type and applied them to the respective combinations to impute missing values.

Concentration ratios (CRs) were used as a measure of how concentrated a promotion factor was within each market segment (combination of the 4 prescriber types and 4 payer types) and US Census region compared with that observed nationally among all prescribers and payers combined. First, the 3 market segments accounting for the highest number of fills among adults aged 18 to 64 or 65 or older were identified at the national level. CRs were then calculated by dividing the medication adherence promotion factor value observed for those 3 market segments within each region by the value observed nationally. For example, a CR was calculated for the percentage of fills acquired via mail order (an adherence promoter) among patients aged 18 to 64 in the South Census region who had a primary care prescriber and Medicaid coverage (market segment) divided by the overall percentage of fills acquired via mail order observed nationally. CRs greater than 1.0 imply an overall higher concentration of that factor within that specific market segment and US Census region compared with what is observed nationally among that age group.

Analyses were conducted in SAS version 9.4 (SAS Institute, Inc). The Human Subjects Review Board of the Centers for Disease Control and Prevention (CDC) deemed use of these de-identified, aggregate data exempt from institutional review board review.

## Results

In 2017, 706.5 million BP medication prescriptions were filled, representing approximately $29.0 billion in total spending, including $4.9 billion in patient spending (Table 1). PCPs were the most frequent prescribers (59.7% of all fills) and commercial insurance the most frequent payer (46.0%). Patients aged 18 to 64 accounted for most fills (52.6%) and patient spending (51.0%), although patients aged 65 or older accounted for most total spending (53.9%).

**Table 1:** Prescription Blood Pressure Medication Fills, Total Spending, and Patient Spending Among Adults Aged 18 Years or Older, by Age Group, Prescriber Type, Payer Type, and US Census Region^a^, 2017

Variable	Fills					Total Spending					Patient Spending				
	US	US Census Region				US	US Census Region				US	US Census Region			
		NE	MW	S	W		NE	MW	S	W		NE	MW	S	W
**Total no.^b^**	706.5	162.1	141.4	285.8	117.2	29.0	6.0	7.0	11.6	4.4	4.9	1.0	1.1	2.1	0.7
**Percentage of Total**															
**Age group, y**															
18–64	52.6	50.0	52.2	53.7	53.6	46.1	43.8	45.8	48.0	45.0	51.0	45.9	48.9	54.8	49.8
≥65	47.4	50.0	47.8	46.3	46.4	53.9	56.2	54.2	52.0	55.0	49.0	54.1	51.1	45.2	50.2
**Prescriber type**															
Primary care physician^c^	59.7	59.1	63.2	58.7	58.3	61.2	65.4	58.5	60.8	59.2	60.3	58.3	64.4	59.7	58.7
Nurse practitioner or physician assistant	16.3	14.6	15.3	17.1	18.1	12.8	11.3	11.9	13.6	14.3	14.4	12.5	13.8	15.4	15.4
Cardiologist	11.9	13.8	10.6	12.1	10.8	14.8	18.2	12.4	14.6	14.3	13.8	17.3	11.6	13.5	13.3
Other	12.0	12.6	10.9	12.0	12.9	11.2	12.0	10.3	11.0	12.3	11.5	11.9	10.2	11.5	12.6
**Payer type**															
Commercial	46.0	45.5	45.4	47.3	44.0	53.8	58.1	53.5	54.2	47.7	54.1	54.0	52.9	55.6	51.7
Medicare	37.6	37.4	38.6	37.5	36.9	37.6	34.4	37.8	37.9	41.1	30.2	32.8	32.3	27.4	31.4
Medicaid	10.9	13.4	11.1	8.0	14.6	5.0	4.9	5.5	3.7	7.6	2.4	3.0	2.4	2.0	2.8
Patient self-pay	5.5	3.8	4.9	7.1	4.5	3.6	2.7	3.2	4.3	3.7	13.4	10.3	12.4	14.9	14.2

Abbreviations: NE, Northeast; MW, Midwest; S, South; US, United States; W, West.

a Data source, 2017 Symphony Health Integrated Dataverse (15).

b Number of fills is in millions and spending is in billions of US dollars.

c Includes family practice, internal medicine, and osteopathic medicine.

Nationally, 11.9% of all fills were fixed-dose combinations (range, 5.8% [Maine] to 17.9% [Mississippi]), 97.4% were for generic formulations (range, 95.2% [New Jersey] to 98.4% [Massachusetts and Minnesota]), 8.6% were obtained from mail order pharmacies (range, 4.7% [Rhode Island] to 14.5% [Delaware]) and 65.9% had lower or no copayment (range, 56.6% [Utah] to 72.8% [California]) (Table 2). On average, 1 year of therapy for a single BP medication cost patients $50 out of pocket (range, $38 [Hawaii] to $76 [Georgia]), and fills had a mean days’ supply of 51.3 days (range, 43.1 [Arkansas] to 63.8 [Maine]). Fixed-dose combination fill rates were highest in the South (median, 13.8% of all fills; range, 10.7% [Florida] to 17.9% [Mississippi]) and were the lowest in the Northeast (median, 9.3% of all fills; range, 5.8% [Maine and Massachusetts] to 13.2% [New Jersey]). Generic formulation fill rates were high throughout the country. Use of mail order pharmacies was lowest in the South (median, 8.0%; range, 5.2% [Mississippi] to 10.2% [Virginia]) and highest in the Northeast (median, 9.8%; range, 4.7% [Rhode Island] to 14.5% [Delaware]). The South had the highest percentage of fills with lower or no copayment (median, 65.3%; range, 61.1% [Texas] to 70.5% [Louisiana]). In contrast, patient out-of-pocket spending per therapy year was highest in the South (median, $51 per therapy year; range, $43 [Florida] to $76 [Georgia]), driven, at least in part, by the South having the lowest median for mean days’ supply per fill (median, 49.9 days; range, 43.1 [Arkansas] to 59.6 [Maryland]). The West had the lowest patient out-of-pocket spending per therapy year (median, $47; range, $38 [Hawaii] to $54 [Colorado]), and the Northeast had the highest median for mean days’ supply per fill (median, 55.8 days; range, 44.7 [Rhode Island] to 63.8 [Maine]).

**Table 2:** Adherence Promoter Values for Blood Pressure Medication, Nationally and by State with Medians by US Census Region, 2017

Region	State	Fixed-Dose Combination Fills, %	Mean No. of Days’ Supply per Fill	Lower or No Copayment Fills, %	Mail Order Fills, %	Generic Medication Fills,%	Patient Spending, in Millions, US$	Patient Spending per Therapy Year, in Millions, US$	Patient Spending per Therapy Year, in Millions, US$
**United States overall**		11.9	51.3	65.9	8.6	97.4	4,926.6	99.4	49.6

**Northeast**	Regional median	9.3	55.8	64.7	9.8	97.5	41.4	0.9	46.8
	Connecticut	10.2	55.7	67.4	8.2	96.1	61.1	1.2	49.5
	Delaware	12.6	61.0	60.9	14.5	96.7	15.4	0.3	47.8
	Massachusetts	5.8	52.2	67.0	9.7	98.4	106.5	2.4	43.5
	Maine	5.8	63.8	61.6	7.4	98.3	21.6	0.5	41.3
	New Hampshire	6.5	55.8	61.8	12.8	97.6	21.5	0.4	48.1
	New Jersey	13.2	56.1	61.6	12.0	95.2	174.9	3.1	56.8
	New York	11.3	49.8	69.2	8.1	96.9	303.4	6.6	46.0
	Pennsylvania	10.5	50.6	64.9	10.0	97.4	232.3	4.9	47.5
	Rhode Island	8.3	44.7	71.1	4.7	98.2	19.1	0.4	44.6
	Vermont	6.3	62.1	64.6	9.9	97.7	8.6	0.2	39.8

**Midwest**	Regional median	11.4	52.5	64.5	9.4	97.5	80.3	1.8	49.1
	Iowa	11.2	51.5	71.0	7.5	98.0	47.7	1.1	44.4
	Illinois	11.8	53.2	64.7	9.3	97.4	197.1	4.0	49.2
	Indiana	13.6	51.7	61.8	12.4	97.0	122.0	2.4	51.7
	Kansas	11.9	49.8	63.2	8.1	97.1	52.5	1.0	53.7
	Michigan	11.6	56.8	64.4	12.8	97.6	162.2	3.7	43.3
	Minnesota	9.6	60.2	64.3	9.4	98.4	70.7	1.6	44.4
	Missouri	10.8	49.3	65.0	9.5	97.0	110.0	2.2	49.9
	North Dakota	10.0	53.6	58.9	6.5	98.0	13.9	0.3	54.7
	Nebraska	12.6	48.6	64.8	7.6	96.8	33.7	0.6	57.1
	Ohio	12.3	49.6	65.4	12.6	97.4	212.2	4.5	46.8
	South Dakota	10.1	53.3	66.2	8.0	97.7	13.8	0.3	49.1
	Wisconsin	10.0	60.2	62.0	11.2	97.8	89.9	1.9	46.5

**South**	Regional median	13.8	49.9	65.3	8.0	97.5	93.4	1.9	50.5
	Alabama	16.0	53.6	61.2	6.0	97.5	96.2	1.9	50.1
	Arkansas	14.5	43.1	67.5	5.9	97.6	61.3	1.1	54.3
	District of Columbia	12.4	49.4	70.6	5.5	97.3	9.4	0.2	49.3
	Florida	10.7	54.9	70.0	7.6	97.8	296.1	6.8	43.4
	Georgia	14.5	47.0	64.1	5.9	97.6	243.1	3.2	76.3
	Kentucky	11.9	44.6	69.4	8.7	97.5	87.0	1.9	46.3
	Louisiana	14.0	44.2	70.5	8.0	97.2	96.1	1.9	50.7
	Maryland	13.7	59.6	61.3	8.8	97.2	90.6	1.8	49.8
	Mississippi	17.9	43.4	68.1	5.2	97.5	64.4	1.1	56.7
	North Carolina	13.9	50.4	64.9	8.7	97.6	168.0	3.3	50.3
	Oklahoma	12.1	52.5	63.9	6.3	97.0	65.1	1.2	52.8
	South Carolina	15.5	48.7	63.3	8.3	97.5	88.3	1.7	52.3
	Tennessee	13.4	51.1	65.6	8.8	97.1	132.9	2.7	50.0
	Texas	15.1	52.0	61.1	8.1	96.9	431.8	7.3	59.1
	Virginia	13.3	51.1	61.4	10.2	97.4	130.7	2.5	52.5
	West Virginia	11.6	47.3	69.8	8.6	97.6	37.8	0.8	45.2

**West**	Regional median	10.8	52.7	65.1	8.1	97.9	29.8	0.6	46.6
	Alaska	11.0	59.1	64.3	8.1	95.6	8.0	0.2	51.5
	Arizona	9.2	51.5	65.1	8.0	97.6	81.8	1.6	49.6
	California	9.4	47.9	72.8	5.3	97.8	332.8	7.5	44.3
	Colorado	11.7	53.8	59.5	10.0	97.2	52.9	1.0	53.5
	Hawaii	12.3	60.1	61.5	6.5	98.1	12.4	0.3	38.2
	Idaho	11.0	55.2	64.4	7.3	97.9	21.1	0.5	46.6
	Montana	9.6	52.7	66.7	8.7	98.3	14.0	0.3	45.5
	New Mexico	10.4	50.3	68.0	8.1	98.3	22.1	0.5	45.8
	Nevada	11.5	49.8	66.1	8.7	97.8	35.4	0.7	49.5
	Oregon	8.4	51.5	69.5	7.0	98.1	44.8	1.1	42.5
	Utah	14.6	53.8	56.6	7.8	98.0	29.8	0.6	53.3
	Washington	8.1	50.8	68.8	8.9	97.9	73.7	1.7	42.4
	Wyoming	10.8	54.6	58.5	9.0	97.3	8.8	0.2	58.0

More than 50% of all BP medication fills observed nationally were concentrated in the 3 largest market segments (prescriber–payer combinations) for each age group (Table 3). Among adults aged 18 to 64 years, the 3 largest market segments were PCPs and commercial insurance (40.5% of fills), nurse practitioners and physician assistants and commercial insurance (11.8%), and PCPs and Medicaid (8.5%). Among adults aged 65 or older, the 3 largest market segments were PCPs and Medicare (43.0% of fills), PCPs and commercial insurance (14.7%), and cardiologists and Medicare (10.2%).

**Table 3:** Prescription Blood Pressure Medication Fill Market Share by Prescriber Type, Payer Type and Patient Age Group^a^, 2017

Payer and Prescriber Combination	18–64 Years		≥65 Years		All Ages
	Market Share, %	Top 3 Rank^b^	Market Share, %	Top 3 Rank^b^	Market Share, %
**Commercial insurance**					
Primary care physician	40.5	1	14.7	2	28.3
Nurse practitioner or physician assistant	11.8	2	2.6		7.4
Cardiologist	6.4		3.9		5.2
Other	7.8		2.3		5.2
**Medicare**					
Primary care physician	5.7		43.0	1	23.4
Nurse practitioner or physician assistant	2.1		8.8		5.3
Cardiologist	1.0		10.2	3	5.4
Other	1.6		6.8		4.0
**Medicaid**					
Primary care physician	8.5	3	2.3		5.6
Nurse practitioner or physician assistant	4.2		0.5		2.5
Cardiologist	1.3		0.5		0.9
Other	2.4		0.4		1.5
**Patient self-pay**					
Primary care physician	3.8		2.4		3.1
Nurse practitioner or physician assistant	1.7		0.6		1.2
Cardiologist	0.5		0.6		0.5
Other	0.6		0.4		0.6

a Data source: 2017 Symphony Health Integrated Dataverse (15).

b Used to identify the top 3 prescriber and payer combinations (market segments) for each age group to determine the greatest concentration of blood pressure medication fills.

**Figure 1:**
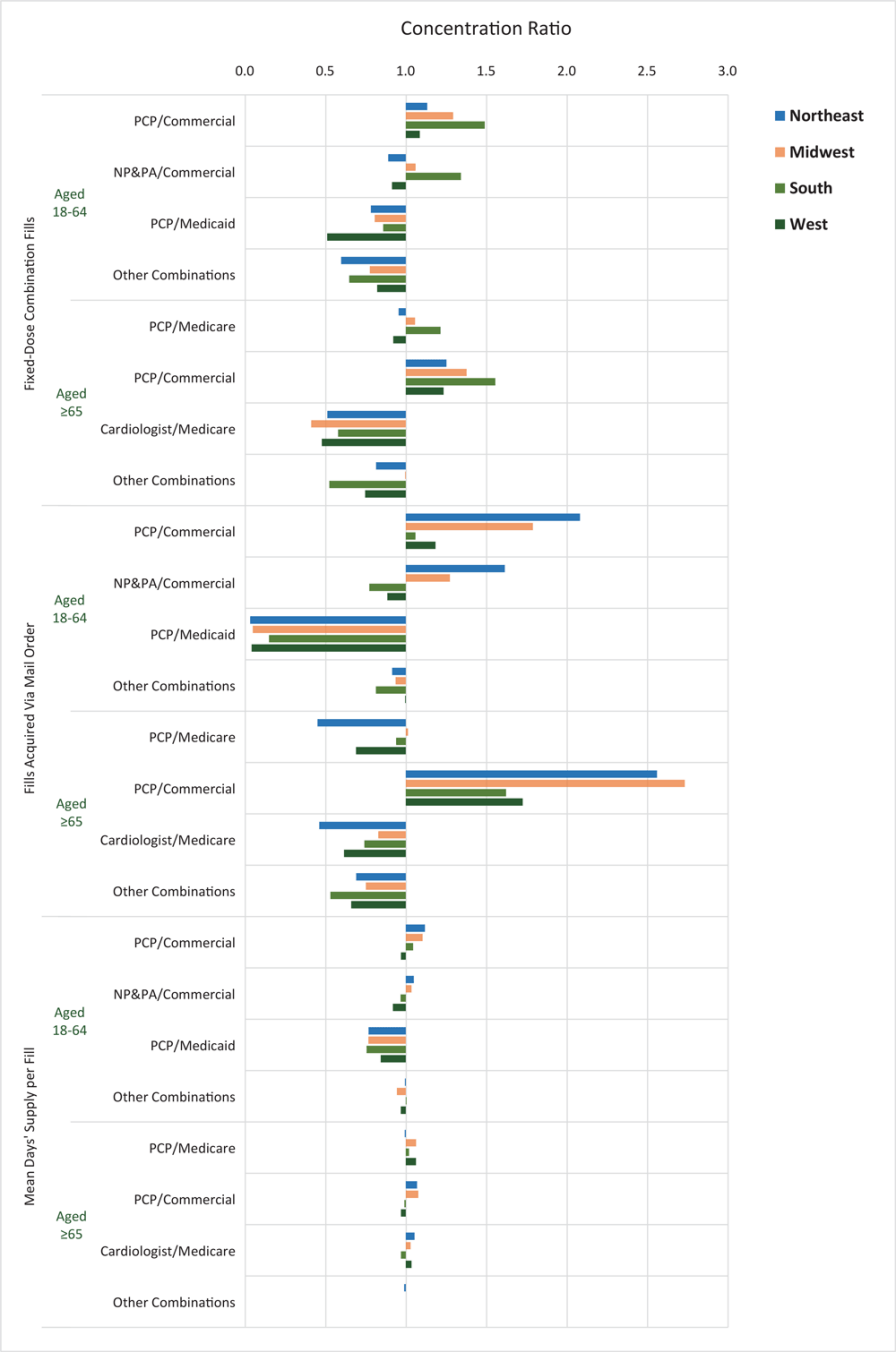
Concentration ratios of adherence promotion factors related to prescriptions among the largest market segments, by US Census region, 2017. Data source: 2017 Symphony Health Integrated Dataverse (15). Abbreviations: NP, nurse practitioner; PA, physician assistant; PCP, primary care physician.

**Figure 2:**
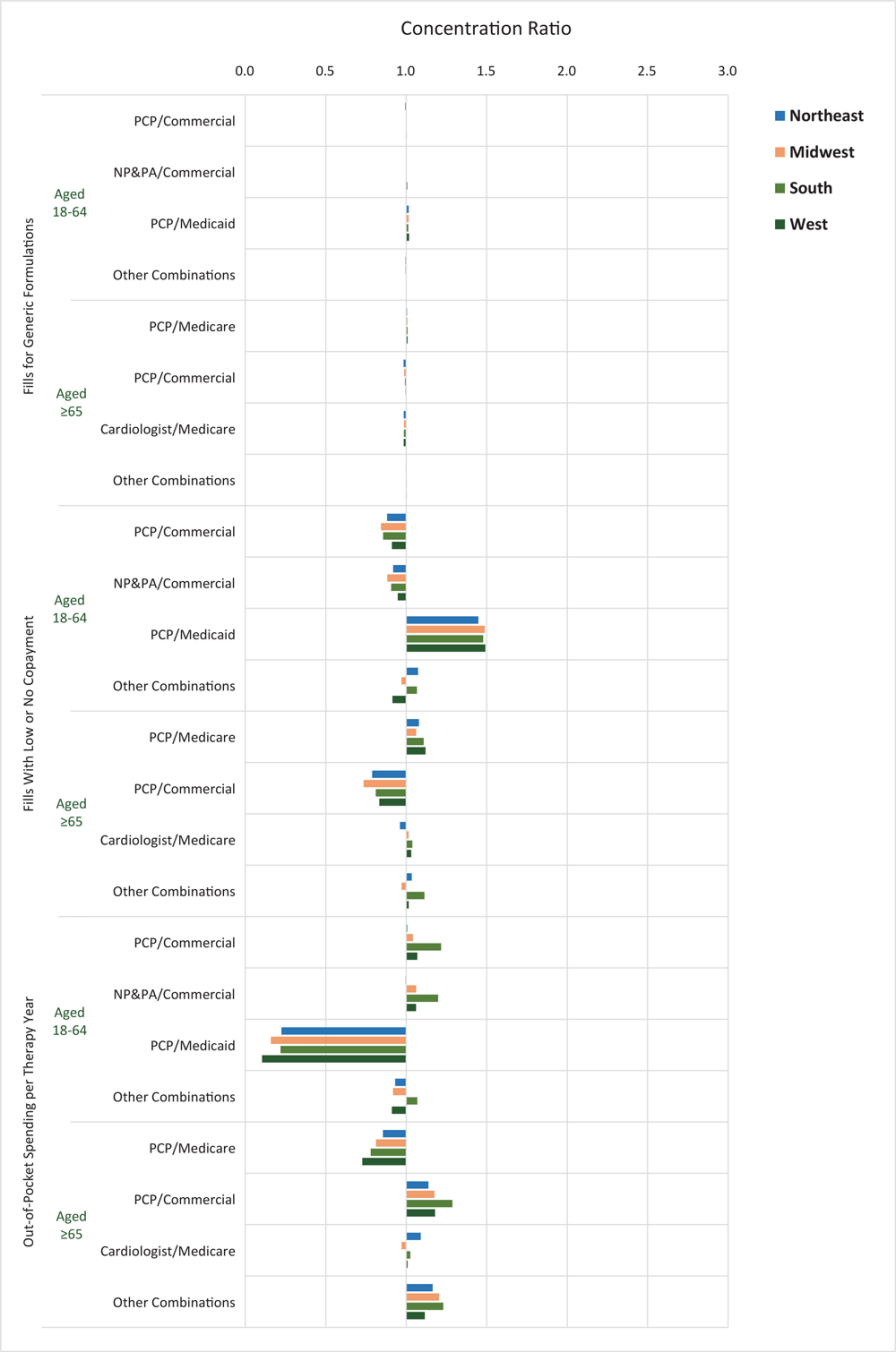
Concentration ratios of adherence promotion factors related to payments among the largest market segments, by US Census region, 2017. Data source: 2017 Symphony Health Integrated Dataverse (15). Abbreviations: NP, nurse practitioner; PA, physician assistant; PCP, primary care physician.

CRs for the prescription-related (Figure 1) and payment-related (Figure 2) adherence promotion factors varied by prescriber–payer combination and US Census region. Fixed-dose combination fills tended to be more concentrated, regardless of age, among patients with commercial insurance compared with public insurance (Medicare or Medicaid), especially in the South (Figure 1). The lowest CRs for fixed-dose combination fills were observed among patients aged 18 to 64 with PCP prescribers and Medicaid coverage (CR range, 0.51 [West] to 0.86 [South]) and patients aged 65 or older with cardiologist prescribers and Medicare coverage (CR range, 0.41 [Midwest] to 0.58 [South]). Mail order fills were most concentrated among commercially insured patients aged 18 to 64 with PCP prescribers across all regions (CR range, 1.06 [South] to 2.09 [Northeast]) or with NP or PA prescribers in the Midwest (CR, 1.28) and Northeast (CR, 1.62), and, among commercially insured patients aged 65 or older with PCP prescribers, across all regions (CR range, 1.62 [South] to 2.74 [Midwest]). The lowest mail order concentrations were observed among patients aged 18 to 64 with PCP prescribers and Medicaid coverage (CR range, 0.03 [Northeast] to 0.15 [South]) and among patients aged 65 or older with cardiologist prescribers and Medicare coverage (CR range, 0.46 [Northeast] to 0.83 [Midwest]). Most of the variation in the concentration of days’ supply per fill was observed among patients aged 18 to 64 with PCP prescribers and Medicaid coverage (CR range, 0.76 [South] to 0.84 [West]).

**Table:** 

Promoter	Age Group, Years	Market Segment	Northeast	Midwest	South	West
Fixed-dose combination fills	18-64	PCP/Commercial	1.13	1.30	1.49	1.09
		NP and PA/Commercial	0.89	1.06	1.34	0.91
		PCP/Medicaid	0.78	0.81	0.86	0.51
		Other Combinations	0.60	0.78	0.65	0.82
	≥65	PCP/Medicare	0.96	1.06	1.22	0.92
		PCP/Commercial	1.25	1.38	1.56	1.24
		Cardiologist/Medicare	0.51	0.41	0.58	0.48
		Other Combinations	0.81	1.00	0.52	0.75
Fills acquired via mail order	18-64	PCP/Commercial	2.09	1.79	1.06	1.19
		NP and PA/Commercial	1.62	1.28	0.77	0.89
		PCP/Medicaid	0.03	0.05	0.15	0.04
		Other Combinations	0.92	0.94	0.81	1.00
	≥65	PCP/Medicare	0.45	1.01	0.94	0.69
		PCP/Commercial	2.57	2.74	1.62	1.73
		Cardiologist/Medicare	0.46	0.83	0.74	0.62
		Other Combinations	0.69	0.75	0.53	0.66
Days' supply per fill, mean	18-64	PCP/Commercial	1.12	1.11	1.05	0.97
		NP and PA/Commercial	1.05	1.04	0.97	0.92
		PCP/Medicaid	0.77	0.77	0.76	0.84
		Other Combinations	0.99	0.94	1.00	0.97
	≥65	PCP/Medicare	0.99	1.07	1.02	1.06
		PCP/Commercial	1.07	1.08	0.99	0.97
		Cardiologist/Medicare	1.05	1.03	0.97	1.04
		Other Combinations	0.99	0.92	0.95	0.94

**Table:** 

Promoter	Age Group, Years	Market Segment	Northeast	Midwest	South	West
Fills for generic formulations	18-64	PCP/Commercial	0.99	1.00	1.00	1.00
		NP and PA/Commercial	1.00	1.00	1.00	1.01
		PCP/Medicaid	1.02	1.02	1.01	1.02
		Other Combinations	0.99	0.99	1.00	1.00
	≥65	PCP/Medicare	1.01	1.01	1.01	1.01
		PCP/Commercial	0.98	0.99	0.99	1.00
		Cardiologist/Medicare	0.98	0.99	0.99	0.98
		Other Combinations	1.00	1.00	1.00	1.00
Fills with lower or no copayment	18-64	PCP/Commercial	0.88	0.84	0.86	0.91
		NP and PA/Commercial	0.92	0.88	0.91	0.95
		PCP/Medicaid	1.45	1.49	1.48	1.49
		Other Combinations	1.07	0.97	1.07	0.91
	≥65	PCP/Medicare	1.08	1.06	1.11	1.12
		PCP/Commercial	0.79	0.74	0.81	0.83
		Cardiologist/Medicare	0.96	1.02	1.04	1.03
		Other Combinations	1.03	0.97	1.11	1.02
Out-of-pocket spending per therapy year	18-64	PCP/Commercial	1.01	1.04	1.22	1.07
		NP and PA/Commercial	1.00	1.06	1.20	1.06
		PCP/Medicaid	0.23	0.16	0.22	0.10
		Other Combinations	0.93	0.92	1.07	0.91
	≥65	PCP/Medicare	0.86	0.81	0.78	0.73
		PCP/Commercial	1.14	1.18	1.29	1.18
		Cardiologist/Medicare	1.09	0.97	1.03	1.01
		Other Combinations	1.16	1.20	1.23	1.12

The concentration of fills for generic formulations was similar across all markets and regions for both age groups (Figure 2). The concentration of fills with lower or no copayment among patients aged 18 to 64 was highest among patients with PCP prescribers and Medicaid coverage (CR range, 1.45 [Northeast] to 1.49 [Midwest and West]) and lowest among those with PCP prescribers and commercial coverage (CR range, 0.84 [Midwest] to 0.91 [West]). The group with PCP prescribers and Medicaid coverage also had the lowest concentration of out-of-pocket spending per therapy year (CR range, 0.10 [West] to 0.23 [Northeast]), whereas those with PCP prescribers and commercial coverage had the highest concentration (CR range, 1.01 [Northeast] to 1.22 [South]). Among patients aged 65 or older, the concentration of fills with lower or no copayment was highest among patients with PCP prescribers and Medicare coverage (CR range, 1.06 [Midwest] to 1.12 [West]) and was lowest among those with PCP prescribers and commercial insurance (CR range, 0.74 [Midwest] to 0.83 [West]). Likewise, patients aged 65 or older with PCP prescribers and Medicare coverage had the lowest concentration of out-of-pocket spending per therapy year (CR range, 0.73 [West] to 0.86 [Northeast]), whereas PCP prescribers and commercial insurance had the highest (CR range, 1.14 [Northeast] to 1.29 [South]).

## Discussion

Despite the 706.5 million BP medication prescription fills that occurred in the United States in 2017, BP medication adherence (16) and BP control rates (17) are low and may be affected by the level of uptake of the adherence promotion factors assessed in this study (18). These factors include modifying how medications are prescribed (ie, prescription-related factors) and by reducing patients’ out-of-pocket costs to obtain the medication (ie, payment-related factors). We found considerable variation in these factors by geography and across the largest market segments serving younger and older adults. The opportunity to increase the use of these adherence promoters, especially in the regions and populations in most need, could improve hypertension control, decreasing risk for negative cardiovascular events, including myocardial infarction and stroke.

Evidence suggests that adherence may be affected by how medications are prescribed (8–10). For example, most patients with hypertension require more than 1 medication to control their BP (19). Prescribing fixed-dose combinations for patients taking more than one BP medication has been shown to increase patient adherence by reducing the number of prescriptions they need filled and by decreasing the number of pills they need to take each day (8). However, fixed-dose combinations constituted only 12% of all national BP medication fills in 2017. Furthermore, the percentage varied considerably by geography and market segment. This includes low concentrations being observed in the South and West — regions with high rates of nonadherence (20,21) — as well as being particularly low among patients aged 18 to 64 years with PCP prescribers and Medicaid coverage. Enrollees in traditional Medicaid more often have a disability, have low income, and have higher rates of chronic disease than similarly aged people with other insurance types (22), and they traditionally have high rates of nonadherence (23). These high rates can be attributed to multiple factors (24), including limited pharmacy access (25), complex drug regimens, and poor refill consolidation (20). Prescribing fixed-dose combination drugs among this population (8), in addition to using other strategies assessed in this study, including use of mail order pharmacies (9) and increasing the days’ supply per fill (10), may help address these barriers. Furthermore, evidence suggests that expanding insurance formulary restrictions or tier status of certain medications, such as generic fixed-dose combinations, within preferred drug lists (26) and covering 90-day prescriptions (27) and use of mail order pharmacies (9) can help reduce barriers to adherence. Therefore, state Medicaid programs seeking to improve their rates of BP medication adherence can consider such options. In addition, outreach to prescribers on potential barriers to adherence that Medicaid patients may be at high risk for, and outcomes of incorporating these promoters in prescribing habits, including avenues for groups to use fixed dose combinations, could support these efforts (28,29).

Improving the affordability of medications by addressing payment-related adherence factors is another opportunity to increase adherence among patients with hypertension (11–13). Minimal variation was observed in generic medication concentrations across markets, suggesting that access to these lower cost therapies is widespread. However, there was notable variation in fills with lower or no copayment and out-of-pocket spending per therapy year, especially by payer type and by region. Lower out-of-pocket spending was more concentrated in public insurance markets, especially Medicaid, while higher copayments and out-of-pocket costs were observed among patients with commercial plans, especially in the South where our analysis identified the highest rates of out-of-pocket spending per therapy year among the commercially insured in this region. Higher costs may impose a barrier to adherence, particularly for low-income patient populations for whom even low costs can be prohibitive (13), especially when these costs are compounded by complex medication regimens potentially needed for multiple comorbidities (30). These cost-related factors may be a reason for the low adherence rates seen in the South (31) and, consequently, may play a role in the region’s lower BP control rates and higher rates of cardiovascular disease morbidity and mortality than in other census regions (32).

Interventions to address many of the barriers to adherence assessed in this study might require large-scale, collaborative, and long-term quality improvement efforts at multiple levels, including the individual prescriber level (15,26). Health care systems and medical practices could consider incorporating evidence-based strategies that focus on increasing uptake of adherence promotion factors among their prescribers. For example, Kaiser Permanente Northern California improved hypertension control rates by prioritizing generic and fixed-dosed combination drugs as first-line hypertension therapies in their standardized treatment approach (ie, protocol) while using multidisciplinary care teams (19). In Minneapolis–St Paul, Minnesota, BP control rates improved from around 30% to around 70% through collaboration with insurance companies, health care institutions, and government agencies that involved collectively developing and adopting clinical guidelines and shared goals for hypertension treatment (33). Key interventions used in these programs and prescription- and payment-related factors highlighted in our study could be replicated and translated into diverse communities to improve BP control. Furthermore, states can work with insurance underwriters (34) to create environments through health insurance market policies with incentives for adherence-promoting prescriptions, like coverage for mail order fills and low copays. Although these measures may lead to higher costs for insurance companies in the short term, they can ultimately lower costs by preventing hospitalizations for expensive acute events (35).

Our study had potential limitations. First, the indications for why medications are being prescribed and whether patients are actually taking the prescriptions they are filling are unknown. If these factors vary by patient demographics or prescriber–payer combinations, it may affect our comparisons across market segments. Second, the cross-sectional nature of this study and the inability to link prescription fill data at the patient level prevents formally establishing relationships between the promotion factors and adherence rates. However, prior studies have described these relationships (7–13). Third, we estimated fills with unknown copay amounts in proportion to fills where copays were known, possibly redistributing fills to incorrect categories. However, the impact was probably minimal because fills with unknown copays represented less than 3% of fills. Fourth, we might have underestimated patients’ average spending per years’ supply because our data captured only copayment-related spending and no other patient spending, including drug plan premiums and deductibles. Fifth, misclassification of payment source for some fills may have occurred. For example, fills acquired under Medicare Advantage–associated Part D plans may have been classified as having commercial payment sources and not Medicare Part D, thereby underestimating fills paid for by the latter. Sixth, we are unaware of any study assessing the relationship between the magnitude of the concentration ratios presented in this study and health outcomes. Further analyses are needed to identify meaningful cutpoints that can be applied to these ratios to help identify the market segments in most need of intervention. Finally, IDV data do not account for fills obtained through systems with their own outpatient pharmacies (eg, US Department of Veterans’ Affairs, integrated private sector delivery systems, Federally Qualified Health Centers); therefore, regional comparisons may be affected by variation in penetration rates of these systems.

Our study identified considerable variation, by geography and across the largest market segments, in prescription- and payment-related factors that promote adherence to BP medication. Future research on the use of adherence promoters by prescribers and payers may identify additional opportunities for interventions. Continued assessment of these data can help evaluate public and private initiatives aimed at addressing these factors in an effort to improve adherence and optimize hypertension management.
